# UV crosslinked mRNA-binding proteins captured from leaf mesophyll protoplasts

**DOI:** 10.1186/s13007-016-0142-6

**Published:** 2016-11-03

**Authors:** Zhicheng Zhang, Kurt Boonen, Piero Ferrari, Liliane Schoofs, Ewald Janssens, Vera van Noort, Filip Rolland, Koen Geuten

**Affiliations:** 1 Department of Biology, KU Leuven, Kasteelpark Arenberg 31, 3001 Louvain, Belgium; 2 Department of Physics and Astronomy, KU Leuven, Celestijnenlaan 200d, 3001 Louvain, Belgium; 3 Department of Microbial and Molecular Systems, KU Leuven, Kasteelpark Arenberg 22, 3001 Louvain, Belgium

**Keywords:** Messenger RNA-binding proteins, Messenger ribonucleoprotein complexes, *Arabidopsis thaliana* leaf mesophyll protoplasts, In vivo UV crosslinking, mRNA-bound proteome

## Abstract

**Background:**

The complexity of RNA regulation is one of the current frontiers in animal and plant molecular biology research. RNA-binding proteins (RBPs) are characteristically involved in post-transcriptional gene regulation through interaction with RNA. Recently, the mRNA-bound proteome of mammalian cell lines has been successfully cataloged using a new method called interactome capture. This method relies on UV crosslinking of proteins to RNA, purifying the mRNA using complementary oligo-dT beads and identifying the crosslinked proteins using mass spectrometry. We describe here an optimized system of mRNA interactome capture for *Arabidopsis thaliana* leaf mesophyll protoplasts, a cell type often used in functional cellular assays.

**Results:**

We established the conditions for optimal protein yield, namely the amount of starting tissue, the duration of UV irradiation and the effect of UV intensity. We demonstrated high efficiency mRNA-protein pull-down by oligo-d(T)_25_ bead capture. Proteins annotated to have RNA-binding capacity were overrepresented in the obtained medium scale mRNA-bound proteome, indicating the specificity of the method and providing in vivo UV crosslinking experimental evidence for several candidate RBPs from leaf mesophyll protoplasts.

**Conclusions:**

The described method, applied to plant cells, allows identifying proteins as having the capacity to bind mRNA directly. The method can now be scaled and applied to other plant cell types and species to contribute to the comprehensive description of the RBP proteome of plants.

**Electronic supplementary material:**

The online version of this article (doi:10.1186/s13007-016-0142-6) contains supplementary material, which is available to authorized users.

## Background

Eukaryotic cells use post-transcriptional gene regulation (PTGR) to determine the fates of RNAs, including RNA processing, transportation, localization, translation and degradation [1]. These processes are controlled by various RNA-binding proteins (RBPs), which interact with RNAs and form ribonucleoprotein complexes (RNPs). Identifying and characterizing RNPs is therefore critical to understand the regulation of cellular RNA metabolism [2]. When considering different RNA metabolic regulation pathways, post-transcriptional regulation of pre-mature mRNAs is particularly important because of the complexity of the pool of mRNAs, their abundance and the additional complexity of translating one or more different protein isoforms from a single gene locus [3].

RBP binding specificities from mainly mammalian cells have been experimentally studied by use of common in vitro methods such as RNA electrophoretic mobility shift assay (REMSA), protein affinity purification, systematic evolution of ligands by exponential enrichment (SELEX), fluorescence methods and nuclear magnetic resonance spectroscopy (NMR) [4–8]. These results have been assembled in an RNA-binding Protein DataBase (RBPDB), which provides us with a comprehensive view of the functions of RNPs, the specificities of RNA-binding domains (RBDs) and the RNA motifs they target [9]. More recently, the first genome-wide mRNA-bound proteome has been characterized for HEK293 and HeLa human cell lines, embryonic stem cells (ESCs) and yeast cells by use of a new experimental strategy called mRNA interactome capture [10–13]. The method entails in vivo UV nucleic acid-protein crosslinking followed by poly(A) tailed mRNA pull-down and protein mass spectrometry (MS). The advantage of UV crosslinking over other types of crosslinking based on chemical fixatives is that it generates covalent bonds specifically between physically interacting proteins and nucleic acids [14, 15]. This allows isolating messenger ribonucleoprotein complexes (mRNPs) from a physiological cellular environment. A recent study has investigated the conservation of the mRNA interactome between yeast and human cells [16]. Interestingly, these authors identified previously unknown but conserved RBPs, suggesting that more proteins have RNA-binding capacities than previously considered. Complementary experimental efforts have been pursued to identify the RNA motifs with which RNA-binding proteins interact through methods such as CLIP or crosslinking and immunoprecipitation. This involves in vivo UV crosslinking, immunoprecipitation and RNA sequencing [10, 13, 16, 17]. Also the RNA-binding sites of UV irradiated RNPs can be detected by a novel approach which combines photo-induced crosslinking, MS and statistical automated analysis [18]. Causal functions of RBPs in plant growth and development have already been clearly established, such as in the regulation of flowering time, in transcriptional regulation of the circadian clock and in the regulation of gene expression in chloroplasts and mitochondria [19–23]. Plant endogenous developmental processes can be tightly integrated with responses to environmental stress, especially to abiotic stress [24]. It is notable that many recent studies have focused on the causal roles of plant RBPs in abiotic stress response, such as salinity, cold, drought or abscisic acid (ABA) signaling [25–28]. In the *Arabidopsis* genome, more than 200 RBP genes have thus far been predicted based on well-defined sequence motifs, such as the RNA recognition motif (RRM) or K homology (KH) domain in the encoded proteins while the number of predicted RBP genes in *Oryza sativa* is approximately 250 [29, 30]. When compared to recent studies of mammalian RBPs, experimental evidence for most of these predicted plant RBPs is mostly missing. Furthermore, many studies used in vitro methods to predict the binding specificities of RBPs and focused on specific RBPs, rather than the entire RBP proteome. The specific RBP association with pre-mRNA in plant cell nuclei by use of in vivo UV crosslinking has been previously reported in Lambermon et al. [31]. Here, we identified in vivo UV crosslinking as a major tool missing from the toolbox to discover RBP proteomes coordinating RNA physiology in plants. Interactome capture is a method that allows the straightforward visual confirmation of the success of UV crosslinking through the observation of a “halo” produced by the captured proteins on the oligo-dT beads and therefore appeared to be a good method to optimize the important parameters for UV crosslinking in plant cells, such as light intensity, irradiation duration and the amount of starting plant material required. We used *Arabidopsis* leaf mesophyll protoplasts (i.e. cells from which the cell wall is removed) as a source material to provide optimal access of UV light to the cells. This cell type has been extensively used to study other cellular processes and is also amenable to transient gene expression protocols to allow rapid functional characterization [32, 33]. Protoplasting is also applicable to other cell types and other plant species (e.g. [34–36]).

## Results and discussion

### mRNA interactome capture from leaf mesophyll cells

**Fig. 1:**
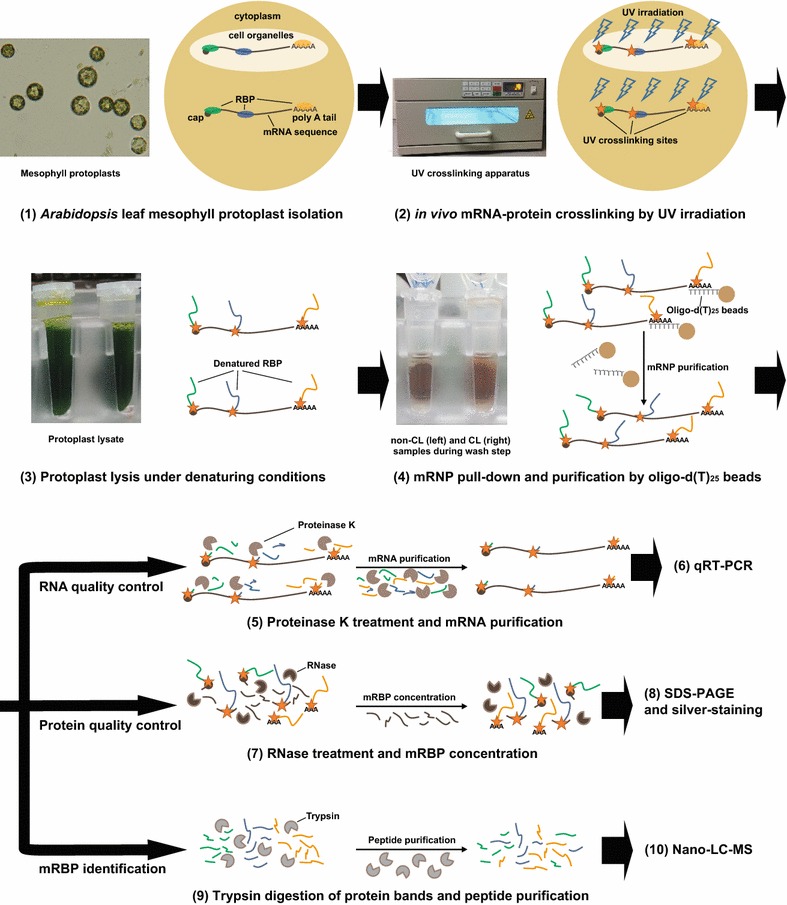
Flowchart of optimized method for discovering mRNA-bound proteome from *Arabidopsis* leaf mesophyll protoplasts. Main steps listed in numbers from 1 to 10. Putative cellular and molecular processes illustrated by cartoons and photos. Details for each step described in “Methods” section

In this study we focus on the mRNA-bound proteome of plant cells, applying the interactome capture method, which was developed for yeast and human cells to plant mesophyll cells, the major type of ground tissue in plant leaves. As illustrated in Fig. 1, the method encompasses ten steps. The first four steps include *Arabidopsis* leaf mesophyll protoplast isolation (1), in vivo mRNA-protein crosslinking by UV irradiation (2), protoplast lysis under denaturing conditions (3) and mRNP pull-down and purification by oligo-d(T)_25_ beads (4). The resulting samples were further analyzed in three ways. RNA quality was checked by proteinase K treatment and mRNA purification (5) followed by qRT-PCR (6). Protein quality control entails RNase treatment and mRBP concentration (7) followed by SDS-PAGE and silver-staining (8). The protein band patterns in the gel are directly compared between a CL sample (in vivo crosslinked mRBPs from UV irradiated protoplasts) and a control sample that was not UV irradiated (non-CL protoplasts as negative control). The final identification of proteins in the CL sample was performed through trypsin digestion of protein bands and peptide purification (9) and Nano-LC–MS (Nano reverse phase liquid chromatography coupled to mass spectrometry assay) (10). Bioinformatic analysis allows identifying mRBPs only present in the CL sample. While the overall procedure is similar to previously reported interactome capture methods for yeast and human cells, some steps had to be modified to be compatible with plant cells.

### Efficiency of mRNA-protein pull-down by oligo-d(T)_25_ beads

**Fig. 2:**
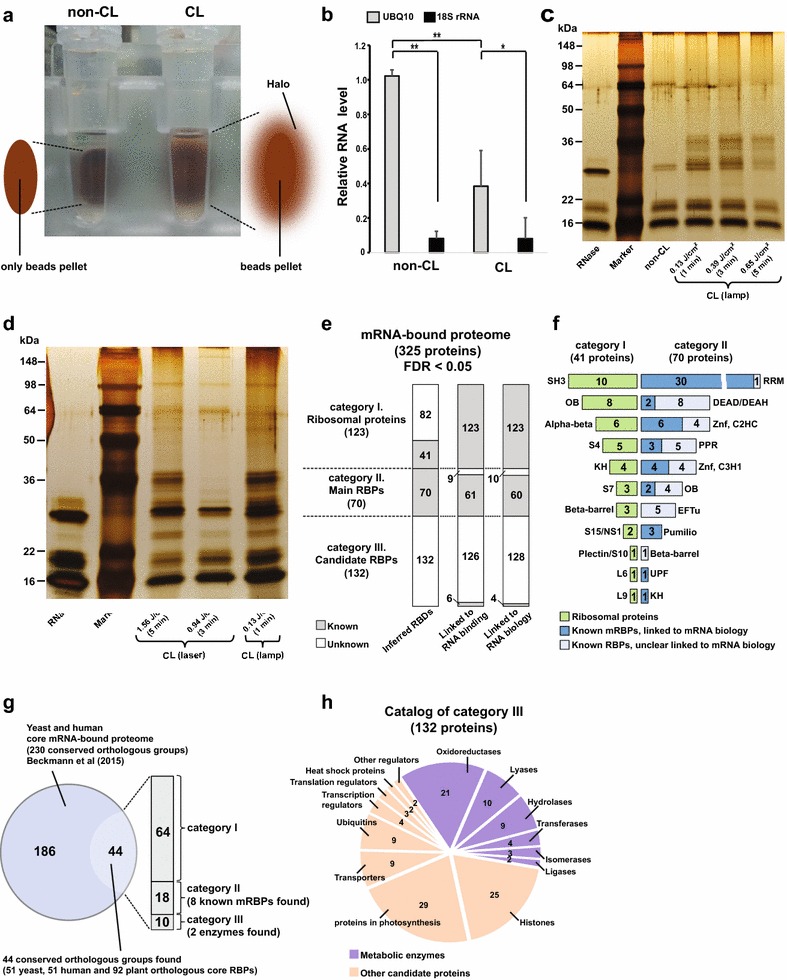
mRNA-bound proteome from *Arabidopsis* leaf mesophyll protoplasts. Observed halo surrounding beads pellet in CL sample and not in non-CL sample during wash step (**a**). 18S rRNA and *UBQ10* mRNA expression levels in non-CL and CL samples by qRT-PCR (values were mean ± SD (n = 3); *single asterisk* and *double asterisk* significant differences with *p* < 0.05 and <0.01) (**b**). Separated mRBPs in protein eluent by SDS-PAGE gels and visualized by silver-staining (**c, d**). Protein eluent of non-CL sample compared with CL samples irradiated by continuous UV for 1, 3 and 5 min (**c**). CL sample irradiated by 1 min continuous UV compared with CL samples irradiated by a pulsed UV laser source for 3 and 5 min (**d**). Classification of three categories from mRNA-bound proteome (quantity of identified proteins listed in numbers and the false discovery rate (FDR) at the peptide and protein levels below 5%) (**e**). List of proteins from category I and II according to the annotated RNA-binding domains (**f**). Detection of plant orthologous core RBPs to yeast and human through comparison between our mRNA-bound proteome and the core mRNA-bound proteome of yeast and human from literature Beckmann et al. [16] (**g**). Pie chart of classification of category III. Quantity of identified metabolic enzymes and other candidate proteins listed in numbers (**h**)

We started by verifying the efficiency of UV crosslinking in plant cells by oligo-d(T)_25_ bead capture. A characteristic halo that surrounds the beads pellet after crosslinking was consistently observed in the CL sample during washing with wash buffer 2 (Fig. 2a). Possibly, this halo is a consequence of bound RNPs that inhibit the dense aggregation of beads through the magnetic field, resulting in a more diffuse aggregation on the magnet. The observation of the halo in the CL sample indicated that pull-down of crosslinked mRNPs by oligo-d(T)_25_ beads was effective [37]. In eukaryotic cells, rRNAs have a higher abundance compared to mature mRNAs [3]. Since oligo-d(T)_25_ beads can only bind poly(A) tailed mRNA, the mRNAs should be enriched in the eluent. In the non-CL control sample, the *UBQ10* reference mRNA is significantly more abundant than 18S rRNA (Fig. 2b). rRNA levels are also low in the CL sample, while mRNA is again significantly enriched. Analysis of the protein samples by SDS-PAGE and silver-staining shows a protein band pattern only present in the CL sample lanes but no specific bands observed in the non-CL control sample that could not be explained by the presence of RNase (Fig. 2c). We conclude that the oligo-d(T)_25_ bead capture is efficient and specific for mRNAs and isolation of mRNPs.

### Optimization of UV crosslinking

We observed that captured proteins in CL samples could only be detected by SDS-PAGE and silver-staining when a minimum of 10^7^ protoplasts is used. Lower concentrations did not yield an observable mRNP pattern on SDS-PAGE and should probably not be used for mass spectrometry because silver-staining and MS detection have similar sensitivity. The duration of UV irradiation and the applied UV light dose is a second critical aspect that determines the efficiency of crosslinking. It is preferable to minimize the duration of irradiation to avoid protoplast damage but sufficient crosslinking still needs to occur. When comparing different UV irradiation times (1–5 min) and UV doses, we obtained protein band patterns in all samples (Fig. 2c). Most optimal was a 1 min UV dose of 0.13 J/cm^2^ as band intensities were indistinguishable between 1 min and 3 min conditions and lower rather than higher staining intensities were observed with a longer crosslinking duration of 5 min of 0.65 J/cm^2^. We finally tested the effect of light intensity and continuous versus pulsed irradiation by replacing the conventional UV lamp with a UV laser source [38]. A pulsed UV laser delivers photons for UV crosslinking in nano-second pulse lengths of 10 Hz and could be more efficient in fixing protein-nucleic acid complexes. We compared samples from 1 min UV lamp irradiation with samples from 3 min and 5 min pulsed UV laser irradiation with UV dose 0.94 and 1.56 J/cm^2^ respectively (Fig. 2d). Again, a similar band pattern appeared when using UV laser irradiation and but the protein yield from the same number of cells appeared lower. As laser irradiation requires a much more complicated experimental setup and does not appear to provide a specific advantage, we propose that a continuous UV source seems to be more optimal for use in standard biological laboratories.

### High abundance of annotated RBPs in the mRNA-bound proteome

Using these optimized conditions, we then set out to analyze the isolated proteins. Identification of proteins was achieved by qualitative and quantitative proteomics (“Methods” section). In qualitative analysis, we identified a total of 341 proteins in CL samples whereas only 8 proteins were detected in the non-CL control samples and 36 proteins were detected in both non-CL and CL samples (Additional file 1: Fig. S1a right). Such enormous difference in the number of identified proteins between non-CL and CL samples is consistent with the previously observed protein band pattern on SDS-PAGE gel (Fig. 2c). For quantitative analysis, protein fold changes (CL/non-CL) based on peptide fold changes from all qualitatively identified proteins were calculated and the results were illustrated in a volcano plot in which all proteins possessing log2-fold changes greater than 2 were considered as positive hits (Additional file 1: Fig. S1a left). From these proteins, there are 225 proteins with log2-fold changes greater than 2, but below the significance level due to data sparsity (only a few peptides present for per protein and the high variability of peptide intensities of low abundant peptides). Because most of them (210 proteins) were qualitatively identified only in CL samples, they were considered as positive hits as well.

In total, we identified 325 proteins in the mesophyll protoplast mRNA-bound proteome (Additional file 1: Fig. S1a). We further classified the proteins into three categories, namely ribosomal proteins (category I), main RBPs (category II) and candidate RBPs (category III) (Fig. 2e), which we annotated within each category by use of Gene Ontology (GO) (“Methods” section). In category I, a high number of ribosomal proteins (123 proteins) was revealed and in category II, a moderate number of classical RBPs (70 proteins) was identified. These two categories indeed represent approximately 38 and 22% of the whole mRNA-bound proteome respectively. The last 40% (132 proteins) were placed into category III since these proteins lack conventional RNA-binding domains and most of their roles in RNA binding or RNA biological processes have not been clarified yet (Fig. 2e, Additional file 2: Table S1). Therefore proteins in this category could reveal novel functions in RNA metabolism. In summary, the interactome capture approach successfully pulled down diverse classes of RBPs from mesophyll protoplasts.

### Most conserved orthologous core RBPs are found as ribosomal proteins from category I

Category I is composed of 101 cytosolic small and large ribosomal proteins (40 and 60S) with a smaller number of 22 chloroplast proteins (30 and 50S). Approximately 33% of ribosomal proteins (41 proteins) possess ribosomal RBDs (Fig. 2e, f, Additional file 2: Table S1). When we mapped the proteins to the core mRNA-bound proteome of yeast and human cells [16], a large number of conserved ribosomal orthologous core RBPs (64 proteins, occupying approximate 52% of category I) was found (Fig. 2g). GO enrichment analysis demonstrated that almost all of ribosomal proteins participate in “gene expression” and “translation” of biological process and they possess a molecular function of “structural constituent of ribosome” (Additional file 3: Table S2), indicating the evolutionary conservation of the putative roles in translation across very distant species.

### Multiple and specific roles of main RBPs in category II

The main RBPs in category II were further classified based on their annotated protein domains known to interact with RNAs. We noticed that a very large number of main RBPs is annotated as linked to RNA binding and RNA biology respectively (Fig. 2e). Furthermore, this category includes 41 proteins considered as “known messenger RNA-binding proteins (known mRBPs)” for which roles in mRNA binding and biology have already been annotated using the GO database (“Methods” section). Another 29 proteins are considered more generally as “known RBPs”, because their role in mRNA processing is not clear yet. When all inferred classical RBDs are listed in Fig. 2f and Additional file 2: Table S1, we noticed that diverse classes of RBDs were discovered. The RNA Recognition Motif domain (RRM) is most abundant in both “known mRBPs” and “known RBPs” groups. Furthermore, it is noteworthy that domain organization is highly diverse. For example, a single RRM domain with repeated copies was identified in series of polyadenylate-binding proteins (AT4G34110, AT1G22760, AT2G23350, AT1G71770, AT1G49760) and multiple RBDs were detected in cold shock protein 2 (AT4G38680), containing OB-fold like domain and zinc fingers. This suggests that different RNA targets could be regulated and RBPs may possess multiple roles in RNA biology. Another example that illustrates this is a group of RBPs which has been experimentally discovered as responding to different abiotic stresses. Schmidt et al. [39] investigated a small *Arabidopsis* mRNA-bound proteome involved in response to reactive oxygen species (ROS) such as hydrogen peroxide. In this study, mRNP pull-down was achieved by oligo(dT) chains on cellulose, somewhat similar to our approach. After comprehensive mapping of mRNA-bound proteomes between our study and Schmidt et al., it is notable that the overlap was significant and included a total of 12 RBPs found in both proteomes (Additional file 1: Fig. S1b, Additional file 2: Table S1). Interestingly, in our category II, 5 RBPs were significantly associated with the specific biological process “response to cold” (AT4G13850, AT2G21660, AT4G39260, AT2G37220, AT4G38680, Additional file 3: Table S2). Because our protoplasts were not under oxidative stress but treated with ice-cold cell culture solution (“Methods” section) and GO annotations of these RBPs refers to “response to cold” or “cold acclimation”, this suggests that the same RBPs were expressed under different abiotic stresses. In contrast to the large number of conserved plant ribosomal core RBPs, we found only 18 orthologs (8 “known mRBPs”) from category II (Fig. 2g). Most of them were significantly enriched in GO annotated “gene expression”, “RNA metabolic process” and “response to cadmium ions” and none of them was related to cold shock stress (Additional file 3: Table S2). This small number suggests distinct roles for RBPs involved in response to environmental abiotic stimuli in plants.

### Diverse biological processes associated with candidate RBPs in category III

A final category including 132 proteins is classified as “candidate RBPs”, similar to the “enigmRBPs” identified in a recent study of mRNA interactomes from yeast and human cells [16]. Notably, most of these yeast and human enigmRBPs are enzymes involved in diverse biological processes and molecular functions, such as glycolysis, protein folding, cell redox homeostasis, ubiquitination or as having kinase activity. In our category III, we found 49 metabolic enzymes, occupying 37% of candidate RBPs while the rest has no annotated enzymatic functions (Fig. 2h, Additional file 2: Table S1). GO enrichment analysis discovered diverse biological processes for these metabolic enzymes, mainly “photosynthesis”, “glycolysis”, “oxidation reduction” and response to environmental stimuli, such as “response to cold”, “response to light stimuli” and “defense response to bacterium” while another 83 candidate RBPs were also involved in other processes, such as “response to heat”, “transmembrane transport” and “nucleosome assembly” (Additional file 3: Table S2). Interestingly, there were 13 metabolic enzymes significantly enriched in “response to cold” (Additional file 3: Table S2), possibly associated with previously discovered RBPs related to “response to cold” from category II. Furthermore, one of these 13 enzymes is the plant ortholog of yeast phosphoglycerate kinase (AT3G12780, Additional file 2: Table S1). RNA-binding capacity of phosphoglycerate kinase has been detected in both yeast and human cells [16], suggesting that plant enzymes could act in RNA metabolism under stress although they lack a conventional RBDs. In the coming years, the role of these metabolic enzymes in RNA biology should be further characterized. Notably, the C-terminal end of ethylene-insensitive protein 2 (EIN2) was recently reported to be cut off in response to ethylene detection and to function in the repression of EIN3-BINDING F-BOX1/2 (EBF1/2) translation through binding of their 3′UTRs in *Arabidopsis* [40, 41]. Our study provides support for EIN2 as a candidate RBP (AT5G03280, Additional file 2: Table S1) with no yeast or human orthologs, suggesting its specific role in direct post-translational regulation of mRNAs in ethylene signaling. Furthermore, mapping to the yeast and human core mRNA-bound proteome indicates that only 10 proteins (2 chloroplast 2-Cys peroxiredoxin enzymes and 8 other candidate proteins) belong to the core RBPs (Fig. 2g, Additional file 2: Table S1). These core RBPs were enriched only in biological processes “response to cadmium ion”, “response to biotic stimulus” and “response to heat” (Additional file 3: Table S2). The small number of orthologs detected in this category indicates that most plant candidate RBPs may serve plant specific functions in RNA metabolism.

## Conclusions

In this study we have successfully developed an efficient mRNA interactome capture protocol that allows inventorying the RNA-binding proteins from plant cells. The advantage of this method is that it specifically identifies proteins with the capacity to physically interact with mRNA in vivo. We have optimized experimental conditions, such as the minimum concentration of cells required for sample preparation, UV irradiation time and source. In addition, we demonstrated the efficiency of mRNP pull-down by oligo-d(T)_25_ bead capture. MS identification of captured proteins confirmed the specificity of our method, as the majority of identified proteins were RBPs that were previously annotated as such in silico. We also present the first experimental evidence in plants for previously unknown RNA-binding activity of protein, with ortholog conserved in yeast cells. Exploring the binding specificities of these candidate RBPs must be continued through other methods, such as CLIP. One example for investigating the binding specificities of a certain RBP to regulate its target mRNA transcript in *Arabidopsis* by use of CLIP, has been demonstrated by literature Zhang et al. [42]. Recently a new article reported the mRNA-bound interactomes from *Arabidopsis* cell cultures and leaf tissue by use of a similar interactome capture approach [43], which highlights the likely importance of this method in the future. Our study differs from that study in that we provide more detailed optimized conditions of the interactome capture approach and focus on leaf mesophyll protoplasts, a single plant cell type. Furthermore, an alternative method, called photoactivatable-ribonucleoside-enhanced crosslinking or PAR-CL (UV-A 365 nm) has also been recommended for investigating mRNA interactomes from yeast and human cells [16, 37]. Our protocol is based on conventional UV crosslinking (cCL), denoting as UV-C 254 nm [44]. PAR-CL needs the incorporation of the photoactivatable nucleotide 4-thiouridine (4sU) into nascent RNAs during RNA metabolisms without toxicity. 4sU is stable when UV-light is absent and has similar base pairing properties as natural uridine. Under UV-A (365 nm) irradiation, 4sU is highly reactive towards to other nucleotides to form covalent bonds with amino acids [15]. Although the efficient uptake of exogenous 4sU into mesophyll protoplasts needs to be later detected by other method which has been previously developed for yeast cell lines [45], future experiments will allow to compare the utility and complementarity of both cCL and PAR-CL approaches.

## Methods

### *Arabidopsis* leaf mesophyll protoplast isolation

Leaf mesophyll protoplasts were isolated essentially as described by Yoo et al. [32] with some modifications. *Arabidopsis thaliana* Col-0 ecotype seeds were soaked in deionized water for 2 days at 4 °C in darkness. Stratified seeds were then sown on a mixture of soil (Peltracom) and vermiculite (Sibli AS) in 50% (v/v). Plant growth conditions were 12 h light/12 h dark cycle at 23 °C with a light intensity of 100 µmol m^−2^ s^−1^ for 4 to 5 weeks. For one (non-CL or CL) sample around 150 fully expanded 2nd or 3th pair true leaves (3–4 per rosette) were cut into 0.5-1 mm strips using a sharp razorblade and immediately transferred and submerged into the enzyme solution in a large Petri dish (150 × 20 mm, SARSTEDT). The 40 mL isotonic enzyme solution contained 400 mM Mannitol, 20 mM KCl, 20 mM MES buffer (pH 5.7), 0.6 g Cellulase R10 (Yakult, Japan) and 0.16 g Macerozyme R10 (Yakult, Japan), supplemented with 10 mM CaCl_2_ and 0.1% (w/v) BSA and was filter sterilized. The Petri dish was covered with aluminum foil and leaf strips were vacuum infiltrated for 30 min and then incubated at room temperature for an additional 2.5 h. From this step, the protoplasts are always kept in darkness. Protoplasts were then released into the enzyme solution by gentle horizontal shaking and the cell suspension was filtered through a 35–75 μm nylon mesh (SEFAR NITEX^®^) using W5 solution (154 mM NaCl, 125 mM CaCl_2_, 5 mM KCl, and 2 mM MES buffer, pH 5.7) to rinse the Petri dish and recover the rest of the cells. Protoplasts were then washed with W5 solution, using centrifugation for 5 min at 100*g*, and gently resuspended in 10 mL W5 buffer yielding approximately 1 × 10^7^ cells from 150 leaves. Protoplasts were then kept on ice for 30 min for recovery and resuspended in 20 mL ice-cold MMg solution (400 mM Mannitol, 15 mM MgCl_2_, and 4 mM MES buffer, pH 5.7).

### In vivo mRNA-protein crosslinking by UV irradiation

Protoplasts of the non-CL sample were kept in MMg solution on ice, while protoplasts of the CL sample were immediately subjected to UV irradiation. For irradiation by the continuous wave 254 nm UV source, the protoplast suspension was transferred into a large Petri dish (150 × 20 mm, SARSTEDT) with addition of an extra 30 mL of ice-cold MMg solution to cover the plate surface. Protoplasts were irradiated at 0.13 J/cm^2^ for 1 min. For irradiation by the pulsed 254 nm UV laser source (Nd:YAG pumped optical parametric oscillator, Quanta-Ray MOPO710, equipped with a BBO crystal based frequency doubling unit), the protoplast suspension was first divided over 6 wells of a multiwell culture plate (35 × 10 mm, Greiner CELLSTAR^®^). The volume in each well was adjusted to 4 mL by adding ice-cold MMg solution. Protoplasts in each well were irradiated by a 35 mm diameter laser beam at 5 mJ/pulse (repetition rate of 10 Hz), giving an average fluence of 0.94 J/cm^2^ for 3 min and 1.56 J/cm^2^ for 5 min. Protoplasts of both samples were collected (combining the cells from the 6 wells), washed an additional one time with 10 mL MMg buffer to remove any remaining digestive enzymes and harvested by centrifugation for 5 min at 100*g*.

### Protoplast lysis under denaturing conditions

The protoplasts of each sample (10^7^ cells) were lysed by adding 9 mL lysis/binding buffer (500 mM LiCl, 0.5% (w/v) Lithium Dodecyl Sulphate (LiDS), 5 mM DTT, 20 mM Tris–HCl, pH 7.5, and 1 mM EDTA, pH 8.0) to the cell pellet resulting in a clear green solution. After homogenization by passing twice through a glass syringe (50 mL, FORTUNA^®^ Optima^®^) with a narrow needle (0.9 × 25 mm, Becton–Dickinson microlance^Tm^ 3) and incubation on ice for 10 min, the lysates were flash-frozen in liquid nitrogen and stored at −80 °C. Samples can be stored for up to 3 weeks.

### mRNP pull-down and purification by oligo-d(T)_25_ beads

All described materials and reagents here are for one non-CL or CL sample. 1.8 mL oligo-d(T)_25_ magnetic beads stock (5 mg/mL, New England BioLabs, cat no. S1419S) was aliquoted into 6 round bottom microcentrifuge tubes (2 mL, SARSTEDT) on ice. In each tube, the beads suspension was mixed with 600 μL lysis/binding buffer using rotation at 4 °C for 2 min. The oligo-d(T)_25_ bead capture involves the following three steps: In the binding step, tubes were first put into a magnetic rack at 4 °C for 3 min resulting in magnetic capture of the beads and clearing of the suspension. After the supernatant was discarded and tubes were removed from the magnetic rack, 9 mL protoplast lysate was aliquoted into these 6 tubes. The whole suspension was then mixed by pipetting followed by gentle rotation at 4 °C for 1 h. In the wash step, the tubes were put back into the magnetic rack at 4 °C for 3 min. The protoplast lysate must be removed by pipetting and kept at 4 °C for an extra two rounds of oligo-d(T)_25_ bead capture. 1.5 mL ice-cold wash buffer 1 (500 mM LiCl, 0.1% (w/v) LiDS, 5 mM DTT, 20 mM Tris–HCl, pH 7.5, and 1 mM EDTA, pH 8.0) was added to the beads in each tube. The beads were resuspended followed by gentle rotation for 1 min. Tubes were then put back into the magnetic rack at 4 °C for 3 min and the supernatant was discarded. This wash step must be repeated once. Afterwards, the same procedure of washing was repeated twice using 1.5 mL ice-cold wash buffer 2 (500 mM LiCl, 5 mM DTT, 20 mM Tris–HCl, pH 7.5, and 1 mM EDTA, pH 8.0) and one time using 1.5 mL ice-cold low salt buffer (200 mM LiCl, 20 mM Tris–HCl, pH 7.5, and 1 mM EDTA, pH 8.0). In the elution step, finally, 500 μL elution buffer (20 mM Tris–HCl, pH 7.5, and 1 mM EDTA, pH 8.0) was added to the beads in each tube. The beads were resuspended and incubated at 50 °C for 3 min to release the poly(A) tailed RNAs. After gently resuspending the beads, tubes were put back into the magnetic rack at 4 °C for 5 min. All eluents (total 3 mL) were be combined into a clean, sterile RNase-free 15 mL conical bottom tube on ice. The quality and quantity of RNAs can be immediately determined. Samples can be frozen in liquid nitrogen and kept at −80 °C for long term storage. The whole procedure was then repeated twice with the stored protoplast lysate (from the first binding step) to deplete poly(A) tailed RNAs, re-using the oligo-d(T)_25_ beads after washing twice with 1 mL ice-cold elution buffer and once with 1 mL ice-cold lysis/binding buffer to adjust the salt LiCl concentration back to 500 mM.

### Proteinase K treatment and mRNA purification

Each non-CL or CL sample yielded a total of 9 mL eluent after three rounds of oligo-d(T)_25_ bead capture step. The RNA concentration of each sample was approximately 10 ng/µL with an *A*_260_/*A*_280_ ratio around 1.9. 1 mL eluent of each sample was taken for RNA quality control. 16 µg Proteinase K (Invitrogen) was added to the eluent to digest the UV crosslinked proteins. After brief vortex mixing, the eluent was incubated at 37 °C for 1 h. RNA was then purified using the InviTrap^®^ Spin Plant RNA Mini Kit (Stratec Molecular).

### qRT-PCR

Efficient synthesis of first-strand cDNA using 1 µg RNA as template was achieved by use of the GoScript™ reverse transcription system (Promega). The sample was diluted to 5 ng/µL with nuclease-free water. cDNA was then amplified and quantified using the GoTaq^®^ qPCR master mix (Promega) and StepOnePlus™ Real-Time PCR cycler (Thermo Fisher) using 10 ng as template. To quantify RNA levels, the comparative Ct method, namely the 2^−ΔΔCt^ method was used [46]. The reference gene here was an endogenous internal control gene *UBQ10* (AT4G05320). qRT-PCR primers for *UBQ10* and 18S rRNA were described in Li et al. [47] and Durut et al. [48].

### RNase treatment and mRBP concentration

Approximately 100 U RNase Cocktail containing RNase A and RNase T1 was added to the remaining 8 mL eluent. One control sample with RNase Cocktail, in which the eluent was replaced by nuclease-free water, was included. After brief vortexing, all samples were incubated at 37 °C for 1 h. After RNase digestion, the eluent was concentrated using Amicon^®^ Ultra‐4 centrifugal filter units (EMD Millipore). After concentration, the end volume of each sample was 100 µL with a total protein yield of approximately 2 µg. Samples can be kept at −80 °C for long term storage.

### SDS-PAGE and silver-staining

25 µL concentrated eluent and a control sample were mixed with 15 µL 2X loading dye and loaded on an SDS‐PAGE gel containing 5% stacking gel and 12% resolving gel including a protein marker (SeeBlue^®^ Plus2 Pre-Stained Standard, Invitrogen). Proteins were condensed at 60 V for 40 min and separated at 160 V for approximately 1 h until the loading dye reached to the end of the resolving gel. Silver-staining of the proteins was performed using the Pierce^®^ Silver Stain Kit (Thermo Scientific).

### Trypsin digestion of protein bands and peptide purification

Gel lanes were hydrated with 50 μL 100 mM NH_4_HCO_3_ for 10 min and dehydrated afterwards with CH_3_CN for 10 min. This was repeated two times and spots were dried afterwards. For enzymatic digestion, gel pieces were covered with 25 μL of a digestion buffer [50 mM NH_4_HCO_3_, 5 mM CaCl_2_, and 6 ng/μL trypsin (Promega)] and incubated on ice for 45 min. The enzymatic digestion was done overnight at 37 °C. The tryptic peptides were extracted in three steps of each 30 min: once with 80 μL of 50 mM NH_4_HCO_3_ and twice with 80 μL of 50% (w/v) CH_3_CN and 5% (v/v) formic acid (FA). The samples were dried and redissolved in 25 μL solution containing 2% (w/v) CH_3_CN and 0.1% (v/v) aqueous trifluoroacetic acid (TFA) and afterwards desalted by use of Millipore Zip Tip µ-C18 columns. The final eluent containing purified peptides was dissolved in 4 μL 60% (w/v) CH_3_CN and 0.1% (v/v) FA and dried again.

### Nano-LC–MS (Nano reverse phase liquid chromatography coupled to mass spectrometry assay)

#### Nano reverse phase liquid chromatography

The LC–MS analysis was performed on a Q Exactive™ Hybrid Quadrupole-Orbitrap™ Mass Spectrometer (Thermo Scientific, San Jose, CA), coupled online to an Ultimate 3000 ultra-high performance liquid chromatography (UHPLC) instrument (Thermo Scientific, San Jose, CA). The UHPLC system was equipped with an Easy Spray Pepmap RSLC C18 column (2 µm particle, 100 Å pore size, and dimensions: 50 µm × 15 cm, Thermo Scientific, San Jose, CA). Before sample separation on the analytical column, the lyophilized sample was resuspended in 16 µL solution containing 2% (v/v) CH_3_CN and 0.1% (v/v) FA solution. Next, 5 µL sample was injected and loaded on an Acclaim Pepmap 100 C18 precolumn (3 µm particle size, 100 Å pore size, nanoviper, and dimensions: 75 µm × 2 cm, Thermo Scientific, San Jose, CA) at a flow rate of 5 μL/min. Sample separation was performed using a 95 min gradient. Mobile phase A consisted of 99.9% H_2_O and 0.1% (v/v) FA and mobile phase B of 19.92% H_2_O, 80% (w/v) CH_3_CN and 0.08% (v/v) FA. Mobile phase B increased from 4 to 10% in 5 min, 10–25% in 50 min, 25–45% in 18 min followed by a steep increase to 95% in 1 min. A flow rate of 300 nL/min was used. An inherent rinse step (10 min gradient, from 4–95% in 5 min) was applied after every 95 min separation gradient.

#### Mass spectrometry assay

The Q Exactive™ Hybrid Quadrupole-Orbitrap™ Mass Spectrometer was operated in data dependent mode. All mass spectra were acquired in the positive ionization mode with an m/z scan range of 400–1600 m/z. For each precursor spectrum, up to the ten most intense ions were selected for the generation of fragmentation spectra. For precursor spectra, a resolving power of 70,000 full width at half maximum (FWHM) was used with an automatic gain control (AGC) target of 3,000,000 ions and a maximum ion injection time (IT) of 256 ms. For fragmentation spectra, a resolving power of 17,000 FWHM was used with an AGC target of 1,000,000 ions and a maximum IT of 64 ms. Dynamic exclusion of 10 s was applied in order to avoid repeated fragmentation of the most abundant ions. Concerning ion selection, a charge exclusion of 1^+^, 6^+^–8^+^ was applied. The raw data from Q Exactive mass spectrometer (.RAW) are available on request.

#### Qualitative proteomics: peptide and protein identification

The Peaks studio software (Version 7, Bioinformatics solutions Inc., Waterloo, ON, Canada) workflow was used to analyze the fragmentation spectra. This software contains four modules: a module for *de novo* sequencing of MS/MS spectra, a Peaks DB search module for database driven peptide identification, a Peaks PTM search module for detection of post-translational modifications and a Peaks Spider search module designed to detect peptide mutations and perform homology search [49–52]. Spectra with the same mass were merged and a default quality threshold of 0.65 was applied. All spectra were searched against the Swiss-Prot database (version December 2013), with the taxonomy set to *Arabidopsis thaliana*. The following search parameters were used: a precursor mass tolerance of 10 ppm using monoisotopic mass and a fragment mass tolerance of 20 mmu. Trypsin was specified as digestion enzyme and maximum 2 missed cleavages were tolerated. Cysteine carbamidomethylation was set as fixed modification, methionine oxidation was set as variable modification. A maximum of 3 variable post-translational modifications was allowed per peptide. Peptide and Protein score thresholds for reliable peptide and protein identification was set such that both had a FDR of <5%.

#### Quantitative proteomics: statistical analysis of mass spectrometry data

Progenesis LC–MS (Nonlinear Dynamics, version 4.1) was used for the label-free quantitative analysis of proteomics data. MS1 peak areas of peptides with 2–8 charges were exported and linked to peptides identified by Peaks studio software by their mass (tolerated error of max. 10 ppm). Afterwards, average log2-fold changes (CL/non-CL) were calculated for each peptide. The fold changes of peptides were grouped by the original protein and evaluated for statistical significance by calculation of *p* values through student *t* test. *p* values were corrected for FDR by the Benjamini-Hochberg method was achieved by use of R language (version 3.3.0). The fold change of a protein was the average of the fold changes of its peptides. The volcano plot was drawn by function package “calibrate” (version 1.7.2) in R language (version 3.3.0) in which the -log10 transformed adjusted *p* values [−log10 (adj. *p* value)] was in function of average log2-fold changes. At last, only proteins were considered as positive hits in our mRNA-bound proteome when they possess the average log2-fold changes greater than 2 with or without significance.

#### Venn diagrams and hypergeometric tests

Venn diagrams to illustrate overlap of mRNA-bound proteomes among three biological replicates or overlap of mRNA-bound proteomes and core mRNA interactomes between our data and the literature were drawn by function package “venneuler” (version 1.1-0) in R language (version 3.3.0). Hypergeometric tests were used to test the significance of overlap by function “phyper” in R language (version 3.3.0). The overlap is significant when the calculated *p* value is lower than 0.05. The *Arabidopsis* proteome was based on “Ara Proteome TAIR10_pep_20110103_representative_gene_model” from TAIR database containing total 27416 proteins, as background for the hypergeometric tests.

#### Catalog of mRNA-bound proteome

A total of 325 identified proteins was classified into three categories based on the items of “molecular functions and biological process” via the Gene Ontology (GO) database and “family and domain” via the InterPro database. Category I or “Ribosomal proteins” contains all detected ribosomal proteins. Proteins from category II or “Main RBPs” were defined as containing annotated protein domains that interact with RNAs or link to RNA binding with known or unknown functions in RNA biology. Furthermore, subgroup “known mRBPs” contains all mRBPs which was defined if they have “mRNA binding [GO:0003729]”, “transcription antitermination factor activity, RNA binding [GO:0001072]”, “mRNA 5′-UTR binding [GO:0048027]”, “mRNA processing [GO:0006397]”, “alternative mRNA splicing, via spliceosome [GO:0000380]”, “mRNA splicing, via spliceosome [GO:0000398]”, “mRNA modification [GO:0016556]”, “mitochondrial mRNA modification [GO:0080156]”, “regulation of translation [GO:0006417]”, “translational initiation [GO:0006413]”, “chloroplast RNA processing [GO:0031425]” in molecular function and/or biological process. Other RBPs belong to subgroup “known RBPs”. Proteins demonstrating known or unknown functions in RNA biology without annotated RNA-binding domains were placed into category III or “Candidate RBPs”. Enzymes from category III were defined based on annotations from the IntEnz database.

#### Definition of plant orthologous core RBPs

Plant core RBPs orthologous to yeast and human were defined via orthologous groups from InParanoid8 dataset [53]. There were total 1933 groups of orthologs containing 5196 *Arabidopsis* (*A. thaliana*) in-paralogs and 2330 yeast (*S. cerevisiae*) in-paralogs. For *Arabidopsis* to human, there were 3119 groups of orthologs containing 7533 *Arabidopsis* (*A. thaliana*) in-paralogs and 5570 human (*H. sapiens*) in-paralogs. The yeast and human core mRNA-bound proteome containing 230 conserved orthologous groups for comparison with our plant proteome was utilized from Beckmann et al. [16].

#### Gene ontology (GO) enrichment analysis

GO enrichment analysis for proteins in each category or orthologous groups was achieved through agriGO (http://bioinfo.cau.edu.cn/agriGO/analysis.php). Database “Arabidopsis genemodel (TAIR9)” was set as a reference. As statistical tests we chose “Fisher” and the Multi-test adjustment method was “Yekutieli (FDR under dependency)” with 0.05 as a significance level.

## Additional files

**Additional file 1: Figure S1.** Identification of mesophyll protoplast mRNA-bound proteome by proteomic analyses. In quantitative analysis (left), the volcano plot displaying the average log2-fold changes (CL/non-CL) and related adjusted *p* values (−log10 (adj. *p* values)) of all proteins. These proteins identified by qualitative analysis and illustrated in venn diagrams (right). Quantity of proteins listed in numbers. Numbers of proteins in the grey frames based on quantitative and qualitative proteomic results considered as positive hits (**a**). Comparison between our mesophyll protoplast mRNA-bound proteome and the small mRNA-bound proteome from literature Schmidt et al. [39]. The hypergeometric test showing the overlapping significance (**b**).

**Additional file 2: Table S1.** Details of mRNA-bound proteome of *Arabidopis* leaf mesophyll protoplasts.

**Additional file 3: Table S2.** GO enrichment analysis for mesophyll protoplast mRNA-bound proteome.
