# Supplementary figures and images for: UV crosslinked mRNA-binding proteins captured from leaf mesophyll protoplasts
Source: Plant Methods. 2016 Nov 3;12:42. doi: 10.1186/s13007-016-0142-6 (PMC5093948; doi:10.1186/s13007-016-0142-6)

**a**

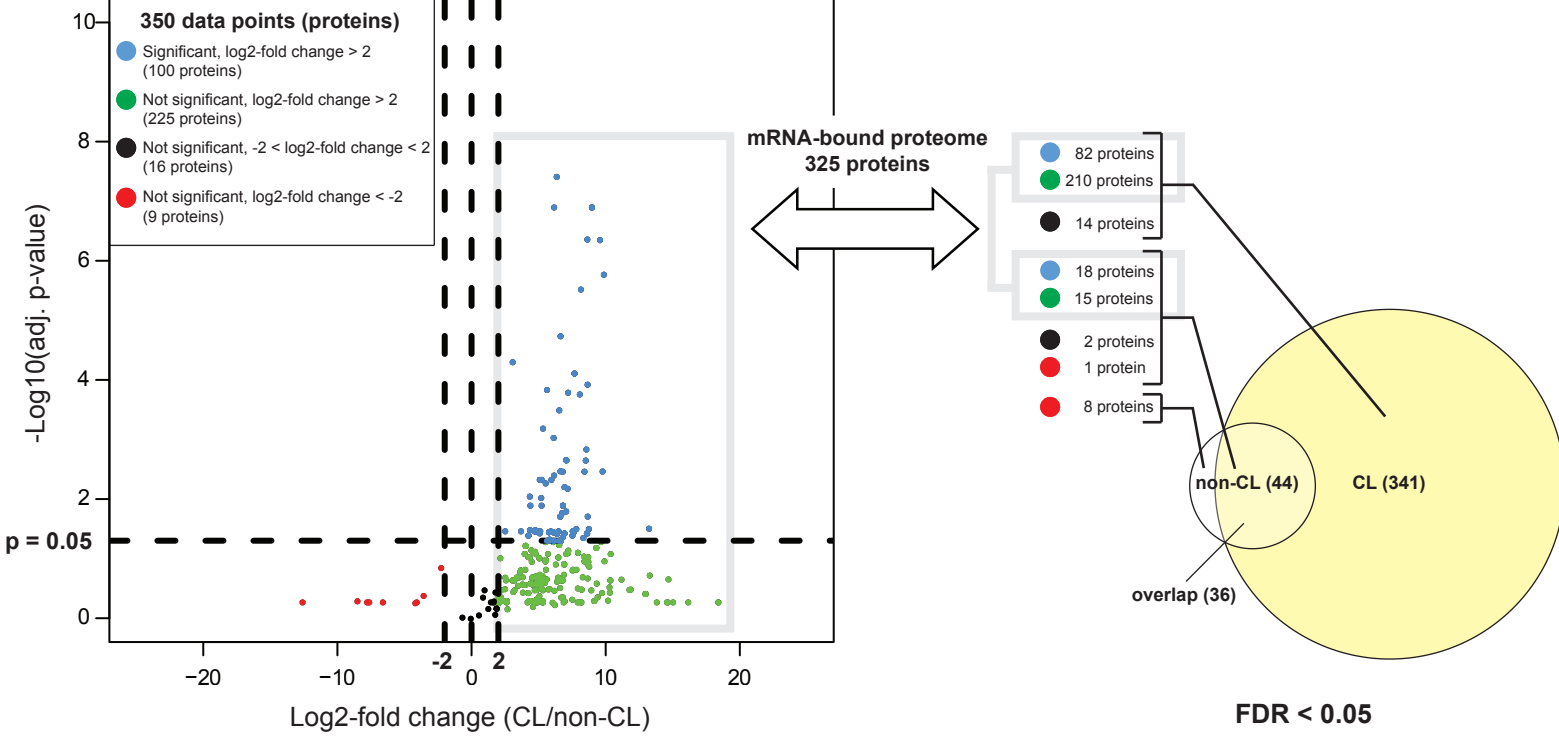

**b**

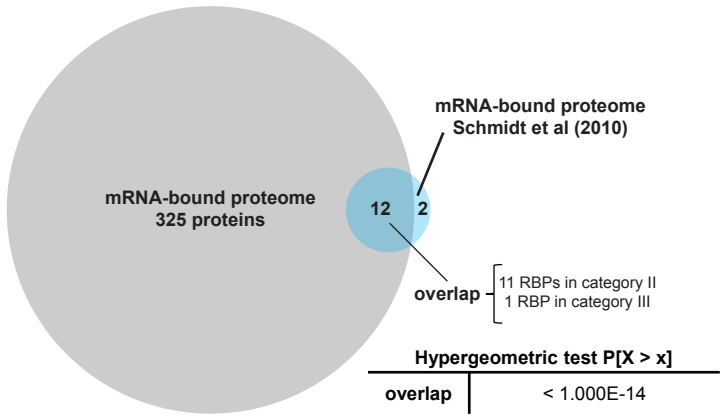

Supplement: Supplementary file 1 — Additional file 1: Figure S1. Identification of mesophyll protoplast mRNA-bound proteome by proteomic analyses. In quantitative analysis (left), the volcano plot displaying the average log2-fold changes (CL/non-CL) and related adjusted p values (−log10 (adj. p values)) of all proteins. These proteins identified by qualitative analysis and illustrated in venn diagrams (right). Quantity of proteins listed in numbers. Numbers of proteins in the grey frames based on quantitative and qualitative proteomic results considered as positive hits (a). Comparison between our mesophyll protoplast mRNA-bound proteome and the small mRNA-bound proteome from literature Schmidt et al. [39]. The hypergeometric test showing the overlapping significance (b). [file 13007_2016_142_MOESM1_ESM.pdf]
